# Research on Cold Start of Proton-Exchange Membrane Fuel Cells Based on Model Predictive Control

**DOI:** 10.3390/membranes13020184

**Published:** 2023-02-02

**Authors:** Shusheng Xiong, Zhankuan Wu, Qi Jiang, Jiahao Zhao, Tianxin Wang, Jianan Deng, Heqing Huang

**Affiliations:** 1 College of Energy Engineering, Zhejiang University, Hangzhou 310027, China; 2 Key Laboratory of Clean Energy and Carbon Neutrality of Zhejiang Province, Hangzhou 310027, China; 3 Jiaxing Research Institute, Zhejiang University, Jiaxing 314050, China; 4 Longquan Industrial Innovation Research Institute, Longquan 323700, China; 5 The Fu Foundation School of Engineering and Applied Science, Columbia University, New York, NY 10027, USA

**Keywords:** fuel cell model, moisture and thermal management, cold start, model predictive control, product water, startup strategies, proton-exchange membrane

## Abstract

The cold start of fuel cells limits their wide application. Since the water produced by fuel cells takes up more space when it freezes, it may affect the internal structure of the stack, causing collapse and densification of the pores inside the catalytic layer. This paper mainly analyzes the influence of different startup strategies on the stack cold start, focusing on the change in the stack temperature and the ice volume fraction of the catalytic layer. When designing a startup strategy, it is important to focus not only on the optimization of the startup time, but also on the principle of minimizing the damage to the stack. A lumped parameter cold-start model was constructed, which was experimentally verified to have a maximum error of 8.9%. On this basis, a model predictive control (MPC) algorithm was used to control the starting current. The MPC cold-start strategy reached the freezing point at 17 s when the startup temperature was −10 °C, which is faster than other startup strategies. Additionally, the time to ice production was controlled to about 20 s. Compared with the potentiostatic strategy and maximum power strategy, MPC is optimal and still has great potential for further optimization.

## 1. Introduction

In recent years, due to the volatility of fossil fuel prices, rising energy demand, and increased awareness of the need for environmental protection, the call for developing fuel cells has increased. Renewable energy such as solar/wind energy is unstable and intermittent in the process of power generation, so the electric energy generated is difficult to use continuously and stably. Therefore, additional use of energy storage/generators is required. In addition, the operation of fuel cell power generation is relatively simple. Fuel cells, especially proton-exchange membrane fuel cells, are pollution-free, noise-free, and have high energy density; thus, they are widely used in vehicles. At present, fuel cell vehicles are widely commercialized. However, the number of hydrogen refueling stations, the cost of fuel cell systems, the safe use of hydrogen, the durability of fuel cells, thermal and water management, and the cold start of fuel cells [1] still limit the wide application of fuel cells. This paper focuses on the cold start of fuel cells. The water produced by the hydrogen–oxygen reaction inside the cell freezes below 0 °C. If more and more ice covers the surface of the catalytic layer or blocks the diffusion layer, preventing the gas from entering the catalytic layer (CL), the stack will cease to be reactive and thus the startup will fail. Since water becomes larger when it turns into ice, it may have an impact on the internal structure of the stack. Common damages [2] include collapse and densification of the pores inside the CL, and pt particle agglomeration and coarsening.

Table 1 describes the current cold-start times of mainstream fuel cell vehicles worldwide [3]. Typical fuel cell models from Toyota and Honda in Japan as well as Hyundai in Korea have achieved a fast cold start at −30 °C within 30 s.

In terms of experimental research on output performance, Li and Wang [4] studied the effects of startup temperature, current density, and microporous layer (MPL) hydrophobicity on the cold-start performance and icing characteristics of a PEMFC through experiments. The research found that the supercooled water in the stack freezes instantaneously at −4 °C and −5 °C, thus reducing the thermal conductivity and heat capacity of the bipolar plate, and that using more hydrophobic MPLs is beneficial for cold-start performance. Jiao [5] et al. designed experiments to study the cold-start process of a PEMFC under different start temperatures and load conditions. The research found that during a potentiostatic startup, fluctuations in cell voltage and current density occur when the internal ohmic resistance of the tested PEMFC is high; during a galvanostatic startup, the current density and temperature distribution are roughly similar to those seen for a potentiostatic start, though the temperature distribution at the end of the galvanostatic start is more uniform due to the increased heat generation rate; it was confirmed that the potentiostatic start is better than the galvanostatic start, and increasing the starting current density is beneficial for the cold start, and finally, three ice formation mechanisms are proposed based on the cathode CL, GDL, and ice layer in the flow channel. 

Huo [6] et al. established an analysis model. In their article, the evolution of water in the cold-start process is classified into four stages: (1) production of cathode water and absorption of membrane water; (2) the water in the ionomer reaches saturation; (3) the water in the ionomer is supersaturated and rapidly turns into liquid and water vapor in the pores; (4) ice formation. The results show that the formation of water in the membrane water state helps to delay the formation of cathode CL ice. The analytical model developed by Mao indicates that dry conditions in the membranes facilitate better cold-start performance by increasing the water uptake and extending the water removal time in the cathode CL.

To study the temperature distribution of a PEMFC during a cold start, Khandelwal et al. [7] established a one-dimensional transient PEMFC stack thermal model to analyze the cold-start capability of a PEMFC and the amount of energy needed during a cold start under different operating conditions and environmental conditions. The model considers the effects of the water–ice phase change and coolant reflux on the cold-start performance of a PEMFC. By analyzing the simulation results, they found that a stack model of 20 cells is sufficient to replace the original stack model, and they also found that for a defined PEMFC stack, there is an optimal operating current density for maximizing the output performance. Mao et al. [8] established an analytical model to explain the electrochemical and thermodynamic behavior of the PEMFC cold-start process, used this model to analyze the cold-start process of a PEMFC at −20 °C and −10 °C, and found that the initial water content and heat capacity of the plate are key parameters affecting the cold start of PEMFC. To study water transfer and ice formation during cold starts, Jiao et al. [9] established a three-dimensional multiphase PEMFC model. The model takes into account the freezing of water in the membrane, the transport of water in the ionic polymer and the catalytic layer, the phase transition of water in the catalytic layer and the gas diffusion layer, and so on. Simulation results show that water freezes first on the cathode catalytic layer, the freezing rate on the anode side is slightly lower, and the anode ice thaws faster. Luo et al. [10] proposed a three-dimensional multiphase PEMFC stack model. In using this model to analyze the cold-start process, it was found that the more cells in the stack, the faster the temperature of the stack rises, the slower the voltage drops, and the easier it is for the stack to achieve a cold start, and the temperature of the cells inside the stack is higher. At the same time, they found that the higher the current density, the closer the ice formed on the cathode catalytic layer to the proton-exchange membrane.

Cold-start schemes can be divided into self-starts and auxiliary cold starts. The most common self-start strategy is galvanostatic startup. Gwak and Ju [11] tried to increase the starting current before the water content of a PEMFC reached saturation, thus avoiding the accumulation of additional ice while increasing the stack temperature, using numerical methods. This method could significantly improve the cold-start performance. Zang and Hao [12] studied the cold-start performance under different current density operation modes and obtained the corresponding current density threshold for a successful startup.

For the cold start of the stack, the output current of the stack is constant in the galvanostatic mode; when the hydrogen–oxygen electrochemical reaction cannot provide enough electrons, it will [13] provide electrons by other means, such as the electrolysis of water, carbon corrosion, and oxidative corrosion of the catalyst, causing damage to the fuel cell structure, which seriously affects its durability. To overcome these problems, Jiang [14] compared the potentiostatic startup with the galvanostatic startup and concluded that the potentiostatic startup has more advantages than the galvanostatic startup in terms of heating time and energy demand. Amamou et al. [15,16] proposed a PEMFC cold-start adaptive strategy based on the maximum power mode, according to the optimization algorithm, to find the maximum power point from the updated model, and then applied the current corresponding to the maximum power point to the PEMFC. In the process, the operating current is controlled in real time to achieve a fast cold start.

The purpose of the various startup schemes and control strategies tried by researchers in the galvanostatic mode, potentiostatic mode, and maximum power mode is to determine the optimal water and heat management scheme, that is, to generate as much water and heat as possible through electrochemical reactions in a short period of time while avoiding a large amount of water accumulation inside the cell after PEM saturation and inhibiting the reaction. This paper intends to design a feedback closed-loop control, and the main strategy used is rapidly increasing the temperature of the stack by increasing the current at the beginning of the cold start, and monitoring the volume fraction of stack ice in real time. In the later stage, the extent of freezing of the reaction product water is reduced by adjusting the current density to ensure the normal progress of the reaction and achieve a balance between water production and heat production.

## 2. Model Simulation

The multidimensional model considers both the effect of time on the variables and the differences inside the stack, such as the temperature, pressure, and flow rate in the XYZ direction at the spatial location, which can indicate the hydrothermal reaction inside the stack more realistically, but the computation time of the model is longer. In this paper, we mainly focus on the strategic optimization of the cold start of the stack, without considering the internal variability. Based on the characteristics of the faster response of the aggregate parameter model, the aggregate parameter model was chosen to build the model.

### 2.1. Stack Model

#### 2.1.1. Empirical Model of the Stack

The empirical model of stack voltage [17] mainly includes the following parts. The theoretical voltage value of a PEMFC is based on the principle of the electrochemical reaction–Nernst equation. The loss caused by the activation of reactor reactants is indicated as the activation voltage drop. The loss caused by concentration differences inside the stack are denoted as the concentration voltage drop. Losses caused by stack internal and plate resistance are indicated as ohmic voltage drop. Due to the existence of impedance, the activation voltage drops, the concentration voltage drops, the ohmic voltage drops, etc. In various parts of the stack, the actual voltage of the PEMFC is lower than the voltage calculated using thermodynamics, and the actual operating voltage can be described by Equation (1):

(1) $$V_{fe}=E_{r}-V_{act}-V_{ohm}-V_{conc}$$

$V_{fe}$ is the actual working voltage, V. $E_{r}$ is the reversible voltage under a non-standard state, V. $V_{act}$ is the activation loss, V. $V_{ohm}$ is the ohmic loss, V. $V_{conc}$ is the concentration loss, V.

The reversible voltage $E^{0}$ under the standard state is:

(2) $$E^{0}=-\frac{\Delta G}{nF}=-\frac{-237000}{2*96400}=1.23V$$

The Nernst equation describes the relationship between reversible electrochemical cell voltage and material concentration, gas pressure, and temperature. The Nernst equation from the reaction of a hydrogen–oxygen fuel cell is:

(3) $$E_{r}=E^{0}+\frac{\Delta S_{0}}{2F}(T-T_{0})-\frac{RT}{2F}\ln\frac{a_{H_{2}O}}{a_{H_{2}}a_{O_{2}}^{0.5}}$$

where R is the ideal gas constant, 8.314 J/(mol·K), T is the temperature, K, a is the representative activity, and $E_{r}$ is the reversible voltage of the fuel cell under the non-standard state, V. $\Delta S_{0}$ is the total reaction entropy change per mole of hydrogen consumed under the standard state, J/K.

The activation voltage drop is the voltage loss caused by the activation of reactants, which then enter the reaction state. Since the water in PEMFCs begins to freeze at 0 °C, the influence of ice on the activation process must be considered, so Zhou [18]’s equation is adopted:

(4) $$V_{act}=-\frac{RT}{2F}\ln(\frac{I}{{\left(1-s_{ice}-s_{lq}\right)}^{0.5}I_{r}})$$

where I is the current density, A/cm^2^, $s_{ice}$ is the volume fraction of ice inside the stack, $s_{lq}$ is the volume fraction of liquid water inside the stack, and $I_{r}$ is the reference current density, A/cm^2^.

The ohmic overvoltage in the stack is described by the following:

(5) $$V_{ohm}=I\times R$$

R is single-cell membrane impedance. Ohmic loss includes electron loss and ion loss. The electron loss between the bipolar plate, the gas diffusion layer and the contact plate is caused by the contact resistance between the fuel cell stack; when the hydrogen ion passes through the electrolyte, the ion loss occurs on the exchange membrane. The main ohmic overpotential of fuel cells mainly comes from ion impedance. This can be determined from the resistance law:

(6) $$R=\frac{\rho \times l}{A_{st}}$$

where ρ is membrane resistivity, being related to the membrane water content, current, and activated area, l is the thickness of the proton-exchange membrane, cm, $A_{st}$ is the activated area of a single cell, cm^2^.

Concentration polarization overvoltage can be expressed as:

(7) $$V_{con}=-\frac{RT}{2F}\ln(1-\frac{I}{I_{\max}})$$

where $I_{\max}$ is the maximum current density, A.

The simulation model of the stack voltage can be obtained according to the above formulas, but the above formulas do not take into account the influence of freezing water or the dynamic temperature change in the PEMFC during startup, which will affect the preparation of the model. Therefore, the state equation and energy transfer equation of water are introduced for the model.

#### 2.1.2. Water Equation of State

According to the direct observation and simulation of water and ice [19], there are various forms of water inside the stack (PEM, CL, GDL). The water not discharged from the stack freezes at low temperatures. When the stack temperature rises due to external heating or irreversible reaction heat itself, the ice melts.

However, the membrane water [20] below the water saturation point will not freeze as the temperature drops due to its tight bond with SO^3−^ radicals, and can always contribute to proton conduction. At the conventional reaction temperature, if the water content in the membrane is saturated, the water will diffuse and transmit in a free state; if it is not saturated, the water [21] will diffuse through the SO^3−^ radicals. During cold initiation, water seeps into some areas of the membrane due to the generation of the reaction product water and the electro-osmotic drag (EOD) effect. If the initial water content of the membrane is very low, the water produced from the infiltrated reaction will become membrane water before the membrane water content reaches the saturation threshold and will not freeze like the initially accumulated membrane water, where the saturation water content of the membrane depends on the membrane temperature. After the water content in the membrane reaches saturation, if the heat generated by the stack reaction is insufficient, in addition to the saturated membrane water freezing, the excess water produced from the reaction [22] will not enter the PEM, and freeze at the interface of the PEM and CL. Theoretically, in the absence of ice nuclei, the liquid phase [23] can maintain a crystallization homogenization temperature of −48.3 °C under standard pressure, and its stability depends on the possibility of ice nuclei formation and growth. At this stage, freezing is a stochastic process. In this paper, regardless of the influence of cold water, we must consider that the excess water will freeze when the temperature is below 0 °C, and the water inside the stack consists of gaseous water, membrane-bound water, and ice. Ice melts above 0 °C, and the internal water includes membrane-bound water, water vapor, and liquid water.

The amount of water generated at the junction of the catalyst layer $n_{H_{2}O}$ and the proton-exchange membrane and stack pressure $P_{w}$ can be obtained according to the following equation:

(8) $$P_{w}=\frac{n_{H_{2}O}RT}{V}$$

*V* is the total volume of the catalytic layer and the proton-exchange membrane.

(9) $$n_{H_{2}O}=\left\{ \begin{array}{l} \frac{\lambda V\rho_{mem}}{EW}+n_{ice},T\leq 0℃\\ \frac{\lambda V\rho_{mem}}{EW}+n_{lq},T>0℃ \end{array}\right.$$

$\rho_{mem}$ is the density of the membrane. EW is the equivalent weight of the proton-exchange membrane. $n_{ice}$ is the amount of ice in the stack, in moles. $n_{lq}$ is the amount of liquid water in the stack, in moles.

#### 2.1.3. Cell Temperature Equation

In an electrochemical reaction, the performance of the stack is extremely related to the temperature, and the success of a cold start also depends on whether the temperature of the stack can be stabilized above 0 °C. Therefore, it is important to establish the temperature model of the stack.

During the reaction of the fuel cell, wherein electricity is released to the external world, remaining energy is given off through heat. The heat change in the stack can be expressed by the equation

(10) $$Q_{cell}=P_{tot}-P_{e}-P_{loss}$$

where $Q_{cell}$ is the heat production of the stack, $P_{tot}$ is the total power of the stack, $P_{e}$ is the output power of the stack, and $P_{loss}$ is the power of the heat exchange between the stack and the outside world.

For a cold start, the warming of the stack is reflected in the power of the stack and the water or ice inside it.

(11) $$Q_{cell}=c_{cell}\frac{dT}{dt}+c_{ice}\frac{dT}{dt}$$

where $c_{cell}$ is the heat capacity of the stack and $c_{ice}$ is the heat capacity of ice. The total power of the stack is related to the degree of electrochemical reactions taking place and can be calculated using the following equation:

(12) $$p_{tot}=1.229I$$

The output power of the stack $P_{e}$ is related to the output voltage of the circuit $V_{fc}$ and can be calculated by the following equation:

(13) $$P_{e}=IV_{fc}$$

The heat dissipated from the stack to the outside world is related to the difference between the temperature of the stack itself and the outside surroundings, which can be calculated using the following equation:

(14) $$P_{loss}=\frac{T-T_{env}}{R_{T}}$$

where $T_{env}$ is the ambient temperature and $R_{T}$ is the thermal resistance.

**Figure 1:**
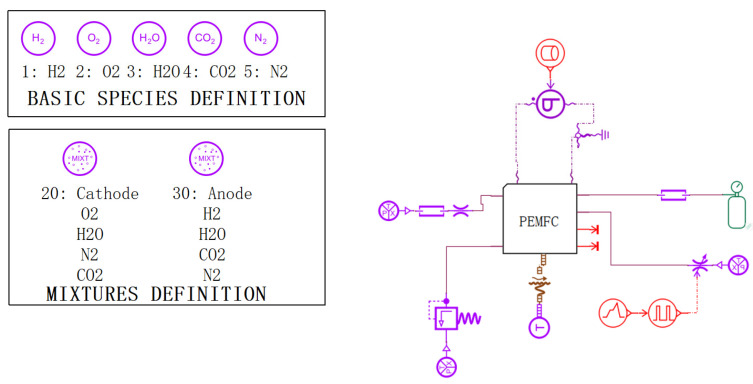
Setting Total parameters for model of fuel cell cold start.

A lumped parameter model of the fuel cell was built by Amesim based on the above equation, as shown in Figure 1.

### 2.2. Model Validation

**Figure 2:**
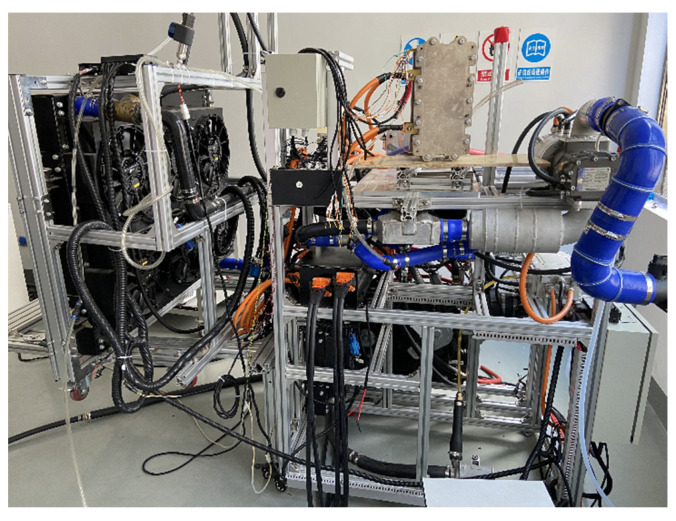
The test bench.

To verify the accuracy of the model, we built a fuel cell test bench. Figure 2 shows the test bench. The initial membrane water content of the stack was 6.4, and the initial temperature was −20 °C. The stoichiometric ratio of cathode to anode was 2.0, the inlet humidity was 0 RH, and the inlet temperature was the same as the initial temperature of the stack. The outlet pressure of the stack was 1 atm. The initial ice volume fraction, initial liquid volume fraction, and initial ice content were all 0.

**Figure 3:**
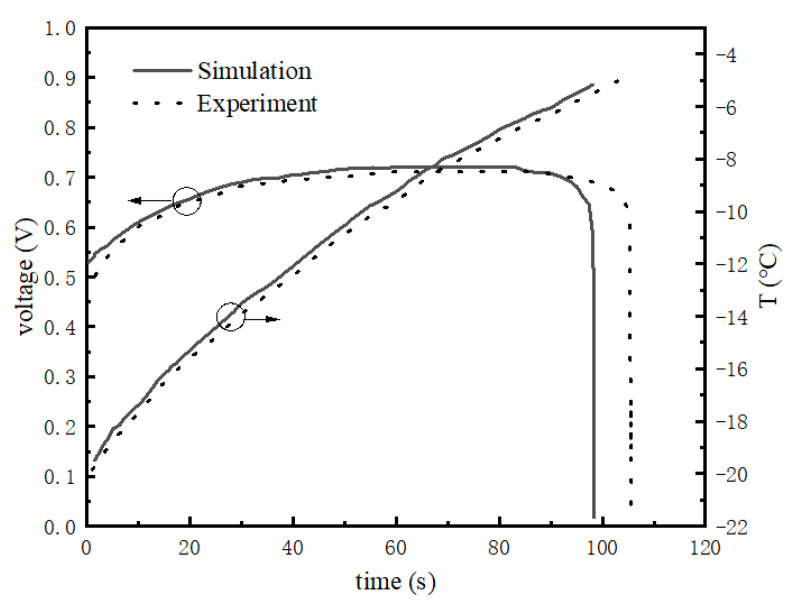
Temperature and voltage variation curve of stack under −20 °C startup.

**Figure 4:**
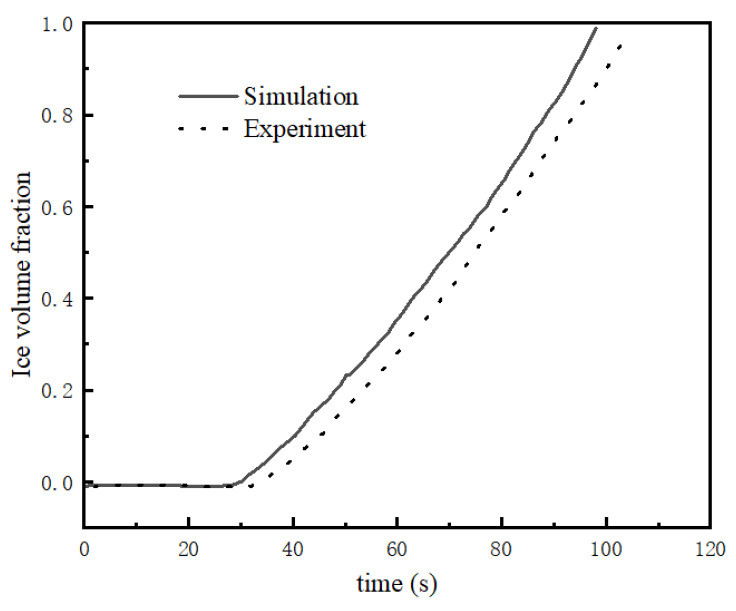
Ice volume fraction variation curve under −20 °C startup.

Figure 3 and Figure 4 shows the difference between the model and experiment at −20 °C and a starting current density of 0.1 A/cm^2^.

Figure 3 and Figure 4 reflect the changes in cell voltage, temperature, and internal ice volume fraction during the startup of the stack at −20 °C. The solid line is the simulated value, and the dotted line is the experimental data. Figure 3 shows the voltage changes in the startup process, which can be divided into three stages, as follows. (1) In the rapidly rising stage (0–35 s), the voltage of the fuel cell rises rapidly with the electrochemical reaction, which is mainly due to the fact that in the initial stage of fuel cell startup, the cell voltage is mainly influenced by the concentration of reactants, and the cell voltage rises rapidly with the increase in reactant concentration. (2) In the slowly rising stage (35–82 s), after a certain period of time at the start, the voltage begins rising slowly. The main reason for this is that after the reactants inside the stack reach a certain concentration, the voltage no longer changes with the increase in reactant concentration, and the temperature becomes the main factor affecting the stack voltage. (3) In the rapid decline stage (82 s-later), the output voltage suddenly decreases rapidly until it reaches zero. During the reaction, water is always generated in the catalytic layer, and will accumulate and freeze, gradually blocking the catalytic layer. Figure 4 shows that at around 28 s, ice began to be produced in the catalytic layer and gradually accumulated. When the volume fraction of ice reaches more than 85%, it will hinder the transfer of reactants, resulting in a drop in output voltage. When the catalytic layer is completely filled with ice, the electrochemical reaction stops and the voltage drops to zero. The maximum error within the effective range was calculated to be 8.9%.

**Figure 5:**
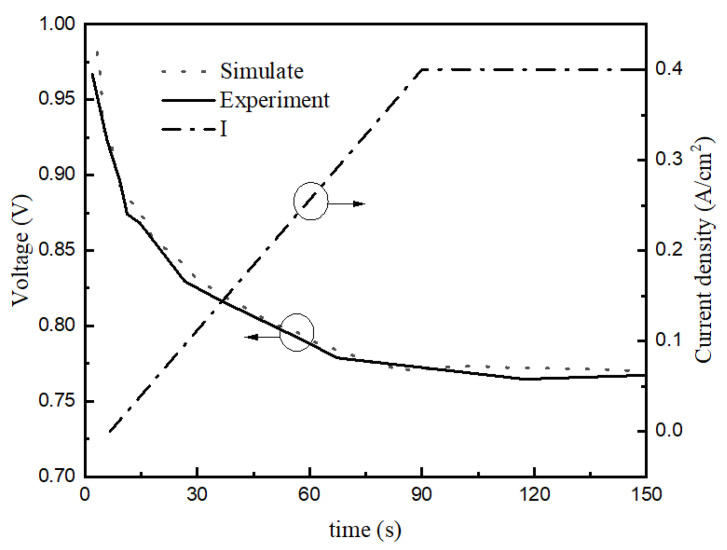
Voltage variation curve of stack under −3 °C startup.

Figure 5 compares the experimental results with the simulation results for a temperature of −3 °C with an initial water content of 6.2. Figure 5 shows that the input current rapidly rose from 0 to 90 s, and the stack voltage decreased with it. From 90 to 120 s, the current was stable at 0.4 A/cm^2^ and the output voltage was also stable without decreasing. The experimental data and the simulation data are basically consistent, with a maximum error of 3.1%.

The results show that the model shows good consistency for both successful and failed low-temperature startup. The model can be considered to meet the requirements and can be used for subsequent studies.

### 2.3. Cold-Start Control Strategy Based on MPC Algorithm 

Model predictive control (MPC), also known as predictive control, is a very important control method in the field of industrial control. The theory of model predictive control [24] is a control method that predicts the future model state in the time domain from the model state of current moment and then solves the optimal control quantity with rolling optimization. MPC is a multivariable control strategy that involves the dynamic model of the loop in the process, the historical value of the control quantity, and an optimal value equation J in the prediction interval. The optimal control quantity can be obtained from the above quantities. 

The most important feature of MPC is that, compared with LQR control, MPC can consider various constraints of spatial state variables, while controls such as LQR and PID can only consider various constraints of input and output variables. MPC can be applied to linear and nonlinear systems.

In order to simplify the calculation, the prediction model used to predict the future state quantity is a simplified model that was built after linearization of the controlled system model, which has the initial state of the current state quantity. The optimal control sequence minimizes the cost function, and the first control sequence of the optimal control sequence is used as the control execution quantity of the controlled system.

The main steps of model prediction are as follows: at the moment k,

1. Estimate or measure the current system state;
2. Carry out optimization calculation based on u_k_, u_k+1_, …, u_k+N_;
3. Take only the value of u_k_ and perform rolling optimization.

Based on the previous subsection, the state equation for the cold start of the fuel cell is determined as Formula (15).

(15) $$\left\{ \begin{array}{l} (C_{sta}+C_{ice})\frac{dT}{dt}=\frac{IA}{2F}\Delta H-IAV_{fc}-\frac{T-T_{env}}{R_{t}}\\ \frac{d\eta_{vice}}{dt}=\frac{\left(\frac{I}{2F}-M_{ice}+M_{out}\right)}{100\rho_{ice}V_{pem}} \end{array}\right.$$

The current I is the input, which represents the startup current. T is the stack temperature and $\eta_{vice}$ is the volume fraction of ice in the catalytic layer, and these two are state variables. The output variables of the system are the state variables. The control system aims to make the input as small as possible so that the system can start with fewer difficulties. It also aims to raise the temperature to 0 °C quickly to minimize the amount of ice left inside the stack. Using Euler’s formula to discretize Equation (15), Equation (16) shows the discretization model, taking *T_s_* as the sampling period.

(16) x(k+1)=Ax(k)+Bu(k)

In the rolling optimization process, the optimal control increment *u*(*k*) of the system needs to be found so that the state vector in the predicted time domain can be close to the reference value. Therefore, this paper takes the performance of the control increment and output error weighting as indicators, and Equation (17) shows the cost function.

(17) $$J=\sum_{i=0}^{N-1}\left(x{\left(k+i|k\right)}^{T}Qx(k+i|k)+u{\left(k+i|k\right)}^{T}Ru(k+i|k)\right)+x{\left(k+N|k\right)}^{T}Fx(k+N|k)$$

We can convert the cost function to quadratic format, as shown in Formula (18).

(18) $$J=x{\left(k\right)}^{T}Gx(k)+U{\left(k\right)}^{T}HU(k)+2x{\left(k\right)}^{T}EU(k)$$

and

$$G=M^{T}\bar{Q}M,\;E=C^{T}\bar{Q}M,\;H=C^{T}\bar{Q}C+\bar{R},$$

$$M=\left[\begin{array}{c} \begin{array}{l} I\\ A \end{array}\\ A^{2}\\ A^{3}\\ \begin{array}{l} .\\ .\\ A^{N} \end{array} \end{array}\right],\;C=[\begin{array}{ccccccc} 0 & 0 & 0 & . & . & . & 0\\ B & 0 & 0 & . & . & . & 0\\ AB & B & 0 & . & . & . & 0\\ A^{2}B & AB & B & . & . & . & 0\\ . & . & . & & & & \\ . & . & . & & & & \\ A^{N-1}B & A^{N-2}B & A^{N-3}B & . & . & . & B \end{array}],$$

$$\bar{Q}=\left[\begin{array}{ccc} Q & \cdots & \\ \vdots & Q & \\ & & F \end{array}\right],\;\bar{R}=\left[\begin{array}{ccc} R & \cdots & \\ \vdots & \ddots & \vdots \\ & \cdots & R \end{array}\right]$$

**Figure 6:**
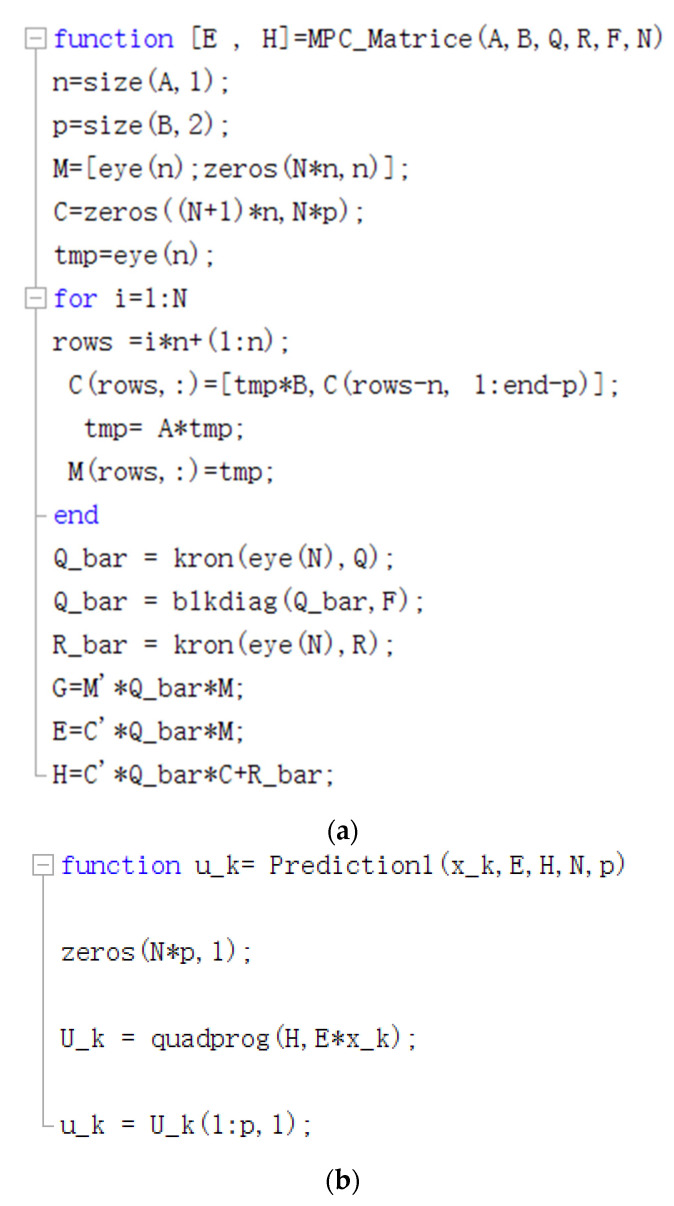
(**a**) MPC algorithm code; (**b**) MPC algorithm code.

**Figure 7:**
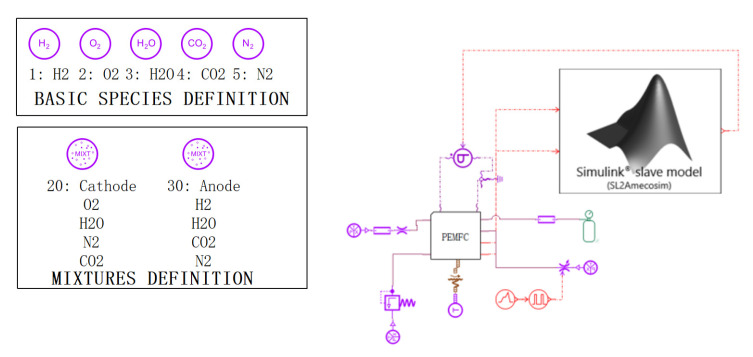
Cold-start model based on MPC control strategy.

The MPC program is written in MATLAB, and Figure 6 shows the key code of the program. The module is imported into AMESIM for joint simulation, and Figure 7 shows the simulation diagram.

## 3. Results and Discussion

To study the effect of fuel cell current density on the cold-start process, the initial water content in the membrane was set to 3, the ambient temperature was −10 °C, and the initial ice volume fraction was 0. The maximum power startup strategy, potentiostatic (0.6 V, 0.45 V, 0.3 V) startup strategy, and MPC-based startup strategy were tested. 

**Figure 8:**
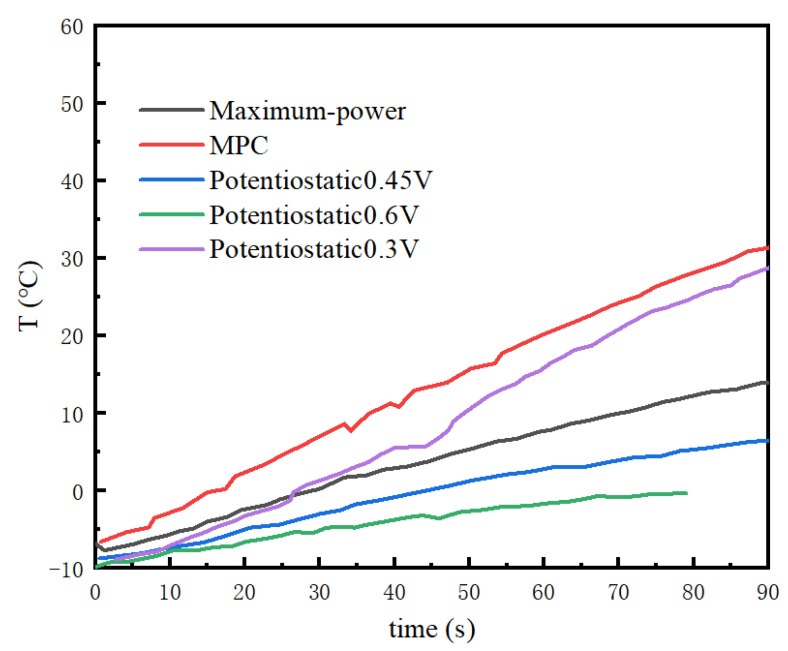
Temperature variation of the stack at −10 °C in different startup modes.

**Figure 9:**
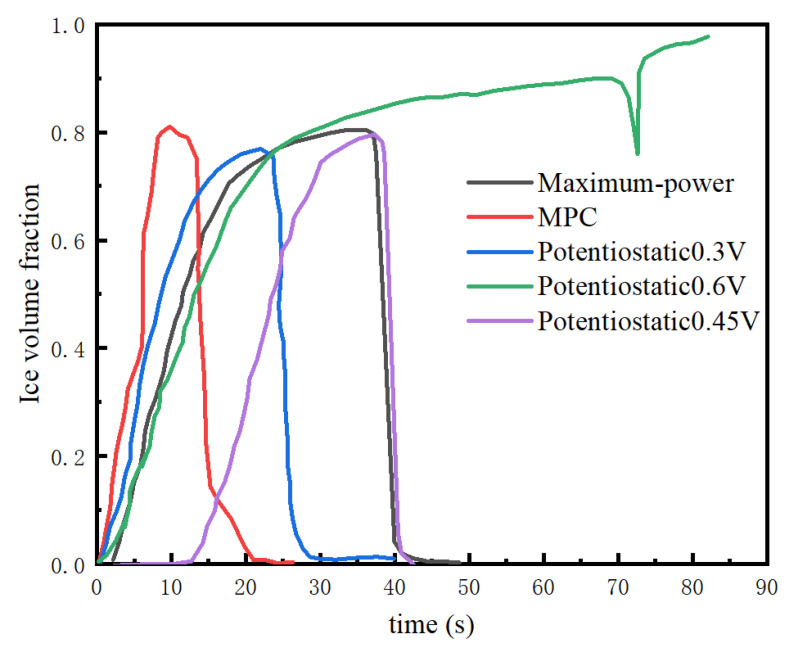
Variation in ice volume fraction in the catalytic layer at −10 °C for different startup modes.

Voltages of 0.3 V, 0.45 V, and 0.6 V were selected comparatively for the potentiostatic startup method. Figure 8 shows the temperature variation at three different startup voltages, and the time needed for the stack to be heated to 0 °C. The 0.3 V potentiostatic startup was initiated 17 s earlier than the 0.45 V potentiostatic startup. When the stack temperature exceeded 0 °C, the temperature rise rate of the 0.3 V potentiostatic startup was also higher than that of the 0.45 V potentiostatic startup. The time needed for the catalytic layer containing ice remained the same for both startup modes, at about 27 s. However, the 0.6 V potentiostatic startup failed. The analysis considering the ice volume fraction in Figure 9 illustrates that when the startup voltage was 0.6 V, the stack temperature was about to rise to the freezing point (70 s to 80 s), and the ice started to melt, triggering a decrease in stack temperature. At this point, the water generated by the reaction continued to turn into ice, blocking the catalytic layer. Additionally, the volume fraction of the ice in the catalytic layer continued to increase until it was completely blocked, and then the cold start of the stack failed.

As for the maximum power startup method, the variations in the stack temperature and the volume fraction of ice in the catalytic layer with time are shown in Figure 8 and Figure 9. The time needed for the stack to reach the freezing point was 28 s, and the stack temperature was 14 °C at 90 s. The volume fraction of the catalytic layer ice during this startup process reached a maximum of 79.5%. From the perspective of temperature rise rate, the effect was not as good as that of the 0.3 V potentiostatic startup strategy and MPC startup strategy. The time to ice production (a duration of 40 s) was the longest in the whole startup process. One the other hand, the maximum power startup mode was not conducive to the rise in the stack temperature.

The MPC cold-start strategy increased the temperature to the freezing point after 17 s, which is faster than the other startup strategies. However, the maximum value of the ice volume fraction in the catalytic layer under the MPC cold-start strategy was the highest, reaching 81%, with the largest ice volume fraction in the catalytic layer. The ice production time was also controlled to be around 20 s, which is the least amount of time among the selected algorithms. Figure 8 shows that the output voltage was always above the safety voltage of 0.1 V, and the stack temperature successfully rose to above 30 °C in 90 s. Overall, MPC was the best among the selected algorithms, while it still had great potential for optimization, such as adjusting the weights of temperature and ice volume fraction and focusing more on the weight of the ice volume fraction in the catalytic layer, which, nevertheless, can lead to a slow rise in the stack temperature. So, the strategy has important significance for cold starts, and when designing startup strategies, we should not only focus on the optimization of the time of the cold start, but also minimize the damage to the stack during the startup process (smaller ice volume fraction and shorter freezing time).

## 4. Summary

Fast and safe startup of PEMFCs at low temperatures is key to ensure proper operation and extend the life of fuel cells. At present, there is no systematic and complete system of water transfer, and the phase change mechanism limits the large-scale commercialization of fuel cells. In this paper, the water distribution, heat transfer, mass transfer, and phase change of a fuel cell during the whole cold-start process are thoroughly investigated by various means, such as experiments and simulations, an optimized fuel cell cold-start model is built, and various cold-start schemes are compared, and the following conclusions were obtained:

1. In comparing the startup modes, it can be found that different startup modes have advantages and disadvantages at different temperatures. However, in general, a higher startup current or a lower startup voltage has a positive effect on the cold start.
2. An MPC-based control algorithm is proposed, which takes the volume fraction and temperature of the catalytic layer of ice as the state variables and the current as the input to adjust the weight coefficients of ice content and temperature. The strategy has been tested and shown to effectively increase the cold-start speed, reduce the ice generation time, and thus avoid damage to the internal structure of the stack. Altogether, the strategy has the potential to optimize the cold start of stacks by minimizing ice and ensuring high heat production.

## Figures and Tables

**Table 1:** Cold-start performance of mainstream fuel cell vehicles in various countries.

Vehicle Type	Country	Era	Output Power (kw)	Temperature (°C)	Time (s)
Toyota Mirai	Japan	2014	114	−30	30
Honda Clarity	Japan	2015	130	−30	30
Hyundai Nexo	Korea	2018	120	−30	30
Roewe 950	China	2014	36	−20	

## Data Availability

Contact the corresponding author.
